# Vocal communication is seasonal in social groups of wild, free-living house mice

**DOI:** 10.1098/rspb.2025.0995

**Published:** 2025-06-18

**Authors:** Nicholas Jourjine, Caspar Goedecker, Barbara König, Anna K. Lindholm

**Affiliations:** ^1^ Department of Organismic and Evolutionary Biology, Harvard University, Cambridge, MA, USA; ^2^ Department of Evolutionary Biology and Environmental Studies, University of Zurich, Zurich, Switzerland

**Keywords:** vocalization, sociality, house mouse, radiotelemetry

## Abstract

House mice (*Mus musculus domesticus*) are among the most widely studied laboratory models of mammalian social behaviour, yet we know relatively little about the ecology of their behaviours in natural environments. Here, we address this gap using radiotelemetry to track social interactions in a population of wild mice over 10 years, from 2013 to 2023, and interpret these interactions in the context of passive acoustic monitoring data collected from August 2022 to November 2023. Using automated vocal detection, we identify 1.3 million individual vocalizations and align them in time with continuously collected telemetry data recording social interactions between individually identifiable mice. We find that vocalization is seasonal and correlated with long-term dynamics in features of social groups. In addition, we find that vocalization is closely associated in time with entrances to and exits from those groups, occurs most often in the presence of pups, and is correlated with how much time pairs of mice spend together. This work identifies seasonal patterns in the vocalizations of wild mice and lays a foundation to investigate the social role of acoustic communication in wild populations of an important laboratory model organism.

## 1. Introduction

Communication between individuals is a fundamental requirement for sociality. Acoustic signalling is one mode of communication that is pervasive among animals, where it has well documented functions in territory maintenance [1] and group cohesion [2], among many others [3]. However, how acoustic signaling affects the dynamics of population-wide social structures remains poorly understood [4]. Understanding this relationship is challenging because it requires monitoring social groups at spatial and temporal scales that are often large (e.g. the area occupied by a population over months or years) while recording acoustic signals taking place at scales that are necessarily small (e.g. the location of an individual as it vocalizes). While advances in hardware [5,6] and software [7–10] have made progress toward overcoming this challenge, the ecologies of many species make it infeasible to simultaneously track social groups and vocal behaviours at the population scale over ecologically meaningful time periods.

House mice (*Mus musculus domesticus*) may be one exception [11]. House mice are human-commensals that typically live at high population density in small areas near human activity, making it feasible to track nearly all individuals in a population in a single location [12]. In the wild and in semi-natural enclosures, they live in social groups [13–15] and are also highly vocal [16,17], producing two major call types that fall into the ultrasonic range (‘USVs’) and human-audible range (‘squeaks’) [18,19]. However, the social role of vocalization in this species has been primarily studied in a limited number of laboratory contexts such as mating [20,21] and neonatal isolation [22]. While this work has uncovered neural mechanisms supporting vocalization in these contexts [23], they are probably a subset of the social contexts in which acoustic communication matters for survival and reproduction in natural habitats. Thus, the role of vocal communication in the social lives of wild mice remains understudied.

Here, we record vocalizations and social interactions in a population of wild house mice living in a barn in the Swiss countryside. Using continuous tracking of individuals as they interact over the course of 10 years, we find that mice occupy seasonally dynamic social groups in which females play a central role, consistent with previous work examining shorter time scales [24]. Using passive acoustic monitoring of the same population from 2022 to 2023, we further find that the amount of time mice spend vocalizing is seasonal, corresponds to events affecting the composition of social groups, and is correlated with how much time pairs of mice spend together. Taken together, these findings identify potential social roles for vocal communication in seasonally dynamic social networks of wild mice.

## 2. Results

**Figure 1.**
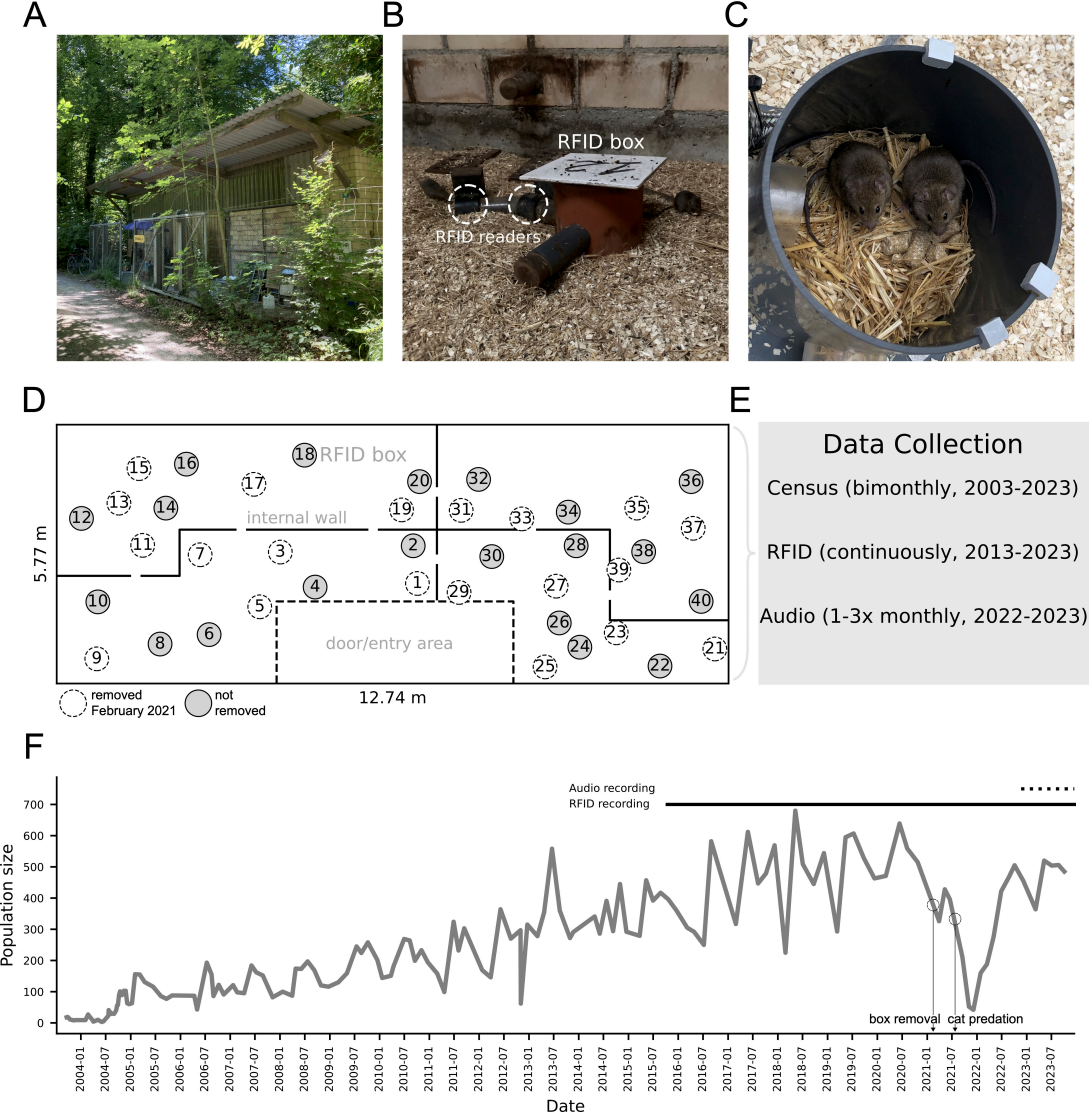
Long-term monitoring of a wild house mouse population. (A) The study site, a barn located at the edge of a forest in an agricultural landscape. (B) An RFID box inside the barn equipped with an entrance tunnel and RFID readers. Note one mouse entering the box (left) while another sits outside (right). (C) Two mice inside an RFID box (lid removed). (D) Barn floor plan with RFID box locations. (E) Data collected. Census: whole population census in which each mouse is caught and phenotyped. RFID: radio frequency identification readings from transpondered mice. Audio: two-day long recordings from individual RFID boxes. (F) Population size in the barn, 2004−2023. The 2021 crash closely followed several cat predation events and an intentional reduction in RFID boxes (as in D).

We focused on a population of house mice (*Mus musculus domesticus*) inhabiting a barn in a wooded agricultural landscape about 20 km from Zürich, Switzerland (figure 1A). The barn contains several small cylindrical boxes each fitted with a single, ground-level entryway and two radio-frequency identification (RFID) readers (‘RFID boxes’, figure 1B,C), in addition to a set of low internal walls and an entry area containing a desk and computer (figure 1D). It provides some protection from predators and contains *ad libitum* food and water, making it an ideal habitat for house mice that recapitulates the human-made infrastructure they typically occupy, while also facilitating long-term monitoring of their behaviour (figure 1E). To this end, twelve mice from neighbouring farms were introduced to the barn in 2002 and have since given rise to a resident population of approximately 400–600 adults. The population experienced one major crash in 2021 caused by a series of cat predation events following the intentional removal of half the RFID boxes (figure 1D,F). Access to the barn is limited only by body size and mice are free to enter and exit. House mice are nonetheless the barn’s only mammalian inhabitant, as confirmed at bimonthly censuses over the last 23 years. During these censuses, all individuals are caught, counted and adults are fitted with a subcutaneous microRFID transponder (Trovan, United Kingdom) if they do not already have one. They are then released back into the barn, where the timestamps of their entrances to and exits from RFID boxes are automatically recorded by a computer system and uploaded to a remote database (see electronic supplementary material, S1 for details).

### (a) RFID box use is season-specific and sex-specific

The barn provides shelter from wind and precipitation but is exposed to seasonal fluctuations in humidity and temperature. As we hypothesized these seasonal changes may be important for understanding social and vocal behaviours, we first analysed RFID readings spanning a 10-year period from 1 January, 2013 to 31 August , 2023, and asked how they were influenced by season, defining ‘spring’ as March, April and May; ‘summer’ as June, July and August; ‘autumn’ as September, October and November; and ‘winter’ of a given year as December of that year plus January and February of the next.

**Figure 2.**
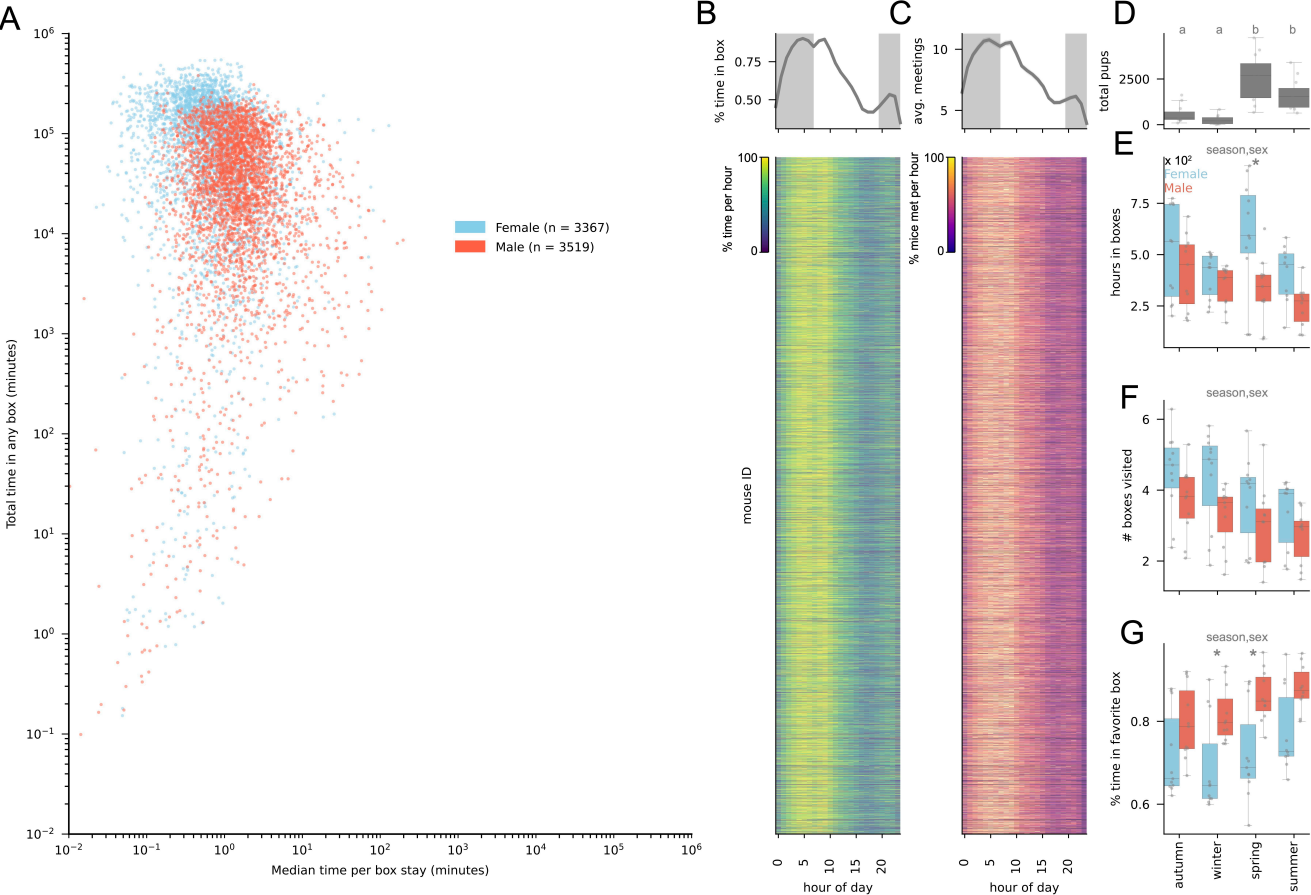
Season- and sex-specific patterns in RFID box use (A) Total time in boxes versus median time per stay for each detected mouse, 2013−2023. Blue: female; red: male. (B) Top: box use by hour of day for each detected mouse, 2013−2023. Shading: night, using average yearly sunrise and sunset for Zürich. Bottom: heat map of data in top plot, one row for each mouse. Colour indicates percentage of each mouse’s time inside boxes at each hour of the day, normalized to the hour with the most time. (C) Top: number of unique meetings with other mice by time of day for each detected mouse, 2013−2023. Bottom: heatmap of data in top plot, one row for each mouse. Colour indicates percentage of each mouse’s meetings at each hour of the day, normalized to the hour with the most meetings. (D) Number of pups discovered by season, 2013−2023, ANOVA with Tukey post hoc test, *p* < 0.001. Letters indicate significantly different seasons. (E) Hours spent in boxes by season and sex, 2013−2023. ANOVA: hours in boxes ~ season × sex. Significant ANOVA predictors are listed above plot (*p* < 0.05). Stars indicate seasons where sex differs significantly by Tukey post hoc test (*p* < 0.05). Each dot corresponds to 1 year. (F) Average number of boxes visited by season and sex, 2013−2023. ANOVA: avg boxes visited ~ season × sex. Significant ANOVA predictors are listed above plot (*p* < 0.05). Each dot corresponds to one year. Within season sex differences by Tukey post hoc test, n.s. (G) Average percentage of time spent in box with most time spent (‘favourite’ box) by season and sex, 2013−2023. ANOVA: % spent time in favourite box ~ season × sex. Significant ANOVA predictors are listed above plot (*p* < 0.05); seasons with significant sex differences are starred. Each dot corresponds to one year. See electronic supplementary material, S2 for complete ANOVA and Tukey tables.

A total of 6946 mice used at least one box in the barn during this time (3519 males, 3367 females, 60 mice with missing sex information), resulting in 39 030 883 individual box stays. An average mouse spent 55 cumulative days inside boxes during 5614 unique stays lasting on average 3 min each (minimum average stay length: 0.02 s; maximum: 23.5 h), all over the course of 149 days from first detected box entrance to last detected exit (figure 2A). Mice, therefore, typically occupied the barn for a period of several months, with many days of cumulative box use consisting of box stays that were short on average but highly variable across mice. While boxes were used throughout the day, box stays increased just before sunrise and after sunset, corresponding to the primary hours of activity for crepuscular house mice [25] (figure 2B). Boxes contained 3.9 mice on average, with the highest occupancies occurring in the early morning (figure 2C). Thus, an average mouse used boxes in the context of social interactions with approximately 3 other mice, consistent with previous work showing boxes are used for breeding and nesting [15,26,27].

As reproduction in the barn population is seasonal, with most pups born in spring and summer (figure 2D; ANOVA: pups ~ season: *p* < 0.001, *F* = 15.4), we asked how box use depended on both time of year and mouse sex. We found that females spent more time in boxes than males, an effect that was driven largely by sex differences in box use in spring (figure 2E; ANOVA: time in boxes ~ season × sex. season: *p* < 0.05, *F* = 2.9; sex: *p* < 0.001, *F* = 13.8; season × sex: n.s.). Females also visited more unique boxes than males, with all mice tending to visit more unique boxes in winter and autumn (figure 2F ANOVA with Tukey post hoc test: # boxes visited ~ season × sex. season: *p* < 0.05, *F* = 3.9; sex: *p* < 0.001, *F* = 12.2; season × sex: n.s.). When mice visited multiple boxes, however, they showed a preference for a single box that was stronger in males than in females, and stronger for all mice in summer (figure 2G; ANOVA with Tukey post hoc test: percentage of time in preferred box ~ season × sex. season: *p* < 0.05, *F* = 3.0; sex: *p* < 0.001, *F* = 31.9; season × sex: n.s.). Thus, males and females differ in their use of RFID boxes, and both change how they use boxes in a season-dependent manner.

### (b) Wild house mice occupy seasonally dynamic social groups

**Figure 3.**
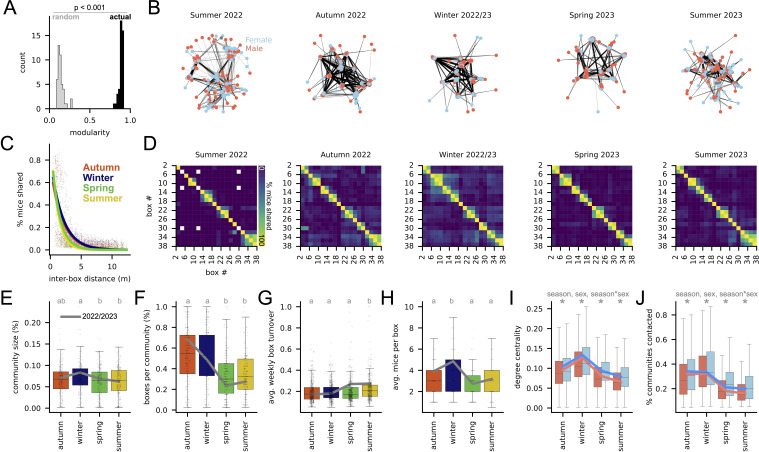
Season and sex-specific patterns in social networks. (A) Network modularity for actual (black) seasonal social networks, 2013−2023, and corresponding randomized networks (grey). Student’s *t*‐test, *p* < 0.001. See electronic supplementary material, S1 for detail. (B) Example social networks, 2022−2023. Nodes are mice; edges connect mice that spent time together; edge length is inversely scaled by how much time, shorter edges for pairs with more time together. See electronic supplementary material, S1 for detail. (C) Mice shared between box pairs (%) versus box pair distance (meters) for each season, 2013−2023. Each dot corresponds to one box pair. Coloured lines are exponential decay models (*y* = *a* × *e*^−*bx*^) fit to data from each season, pooling across years. (D) Examples of mice shared between box pairs for each possible box pair during audio recording period, 2022−2023. White cells correspond to pairs where neither box had inhabitants during the indicated season. Note increased off-diagonal values in autumn and winter. (E) Social network community size (as percentage of total population size) by season, 2013−2023. ANOVA with Tukey post hoc test, *p* < 0.05. Letters indicate significantly different groups. Each dot corresponds to one detected community from one seasonal social network. Grey line connects average values for seasons during which audio was recorded, 2022−2023. (F) Number of unique boxes visited per community, 2013−2023. ANOVA with Tukey post hoc test, *p* < 0.001. Letters indicate significantly different groups. Each dot corresponds to total boxes visited per community per seasonal social network. Grey line as in E. (G) Average weekly change in box occupancy, 2013−2023. ANOVA with Tukey post hoc test, *p* < 0.001. Letters indicate significantly different groups. Each dot corresponds to the average weekly turnover in unique occupants per box per season, 2013−2023. Grey line as in E. (H) Average mice per box, 2013−2023. ANOVA with Tukey post hoc test, *p* < 0.001. Each dot corresponds to the average number of mice per box per season. Letters indicate significantly different groups. Grey line as in E. (I) Degree centrality for male and female nodes in all social network graphs, 2013−2023. ANOVA: centrality ~ season × sex. Significant ANOVA predictors are listed above plot (*p* < 0.001). Stars indicate seasons where sex differs significantly by Tukey post hoc test (*p* < 0.001). Each value represents one individual per seasonal social network. Red line: average male during audio recording, 2022−2023. Blue line: average female during audio recording, 2022−2023. (J) Percentage of total social network communities contacted by each male and female, 2013−2023. ANOVA: percent_contacted ~ season × sex. Significant ANOVA predictors are listed above plot (*p* < 0.001). Stars indicate seasons where sex differs significantly by Tukey post hoc test (*p* < 0.001). Each value represents one individual per seasonal social network. Red and blue lines as in (I). See electronic supplementary material, S2 for complete ANOVA and Tukey tables.

How are these patterns of box use related to population-wide social structure? To address this, we generated social networks from seasonally defined mouse groups in which nodes represent mice and edges represent how much time pairs of mice spent together in any box, normalized to their total box use (their ‘co-occupancy index’, or COI; see electronic supplementary material, S1 for detail.). These social networks were significantly more modular than expected by chance (figure 3A; Student’s *t*‐test. *p* < 0.001, *t* = 84.3), indicating a high level of social structure (see figure 3B, electronic supplementary material, figure S2A for example networks). To better understand this structure, we first asked how groups of mice were distributed among boxes in the barn. We found that boxes that shared mice were typically adjacent and that the distance between these boxes tended to be larger in winter and autumn than in spring and summer (figure 3C; see figure 3D and electronic supplementary material, figure S2C for examples). This suggests that mice occupy spatial territories whose size expands in winter and contracts in summer.

We next partitioned social networks into communities of interacting mice (using the Louvain method; see §4), then asked how features of these communities differed by season over the last ten years. We found that communities tended to be larger (figure 3E; ANOVA: community size as a percentage of total population ~ season: *p* < 0.05, *F* = 3.4) and occupy a larger number of boxes (figure 3F; ANOVA: boxes per community as a percentage of total boxes ~ season: *p* < 0.001, *F* = 31.4), in winters than in summers. Consistent with these trends, boxes experienced less week-to-week turnover in their occupants (figure 3G; ANOVA: average weekly turnover ~ season: *p* < 0.001, *F* = 7.7), and contained more mice (figure 3H; ANOVA: mean no. mice per box ~ season: *p* < 0.001, *F* = 15.9) in winters than in summers. Given their differences in RFID box use, we also asked whether males and females differed in their location within social networks. Across all seasons, females interacted with more mice than males, resulting in higher degree centrality (figure 3I; ANOVA: degree centrality ~ season × sex; season: *p* < 0.001, *F* = 673.7; sex: *p* < 0.001, *F* = 245.9; season × sex: *p* < 0.001, F = 6.2), and were also more likely to interact with mice from communities other than their own (figure 3J; ANOVA with Tukey post hoc test: percentage of total communities contacted ~ season × sex; season: *p* < 0.001, *F* = 948.7; sex: *p* < 0.001, *F* = 170.4; season × sex: *p* < 0.001, *F* = 9.1). Thus, social structure in this population exhibits seasonal dynamics in which males and females play different roles.

The barn population underwent a dramatic decline in 2021 following a series of cat predation events, resulting in the loss of hundreds of individuals (figure 1F). Because our audio recordings were carried out exclusively after this crash, we considered what effect it had on features of social networks in the barn (electronic supplementary material, figure S2B). In contrast to recent studies in primates [28], we did not find dramatic effects of this event on social network structure: neither the number of interactions per mouse (i.e. node density) nor the extent to which the population-wide social network could be divided into communities (i.e. graph modularity) were affected by the crash (electronic supplementary material, figure S2B,D,E). We did find that average node clustering coefficients were higher in social networks following the crash (electronic supplementary material, figure S2F; Student’s *t*‐test, *p* < 0.01, *t* = 3.2), although we cannot exclude that this is due to the intentional reduction in box number that occurred in 2021. With this exception, social structure in the barn population appears to be largely resilient to large changes in population size, consistent with work on shorter time scales following a single predation event [29].

To explore what might explain this resilience, we tested if mouse social networks have ‘small world’ features (i.e. high overall clustering and short average path lengths), which have been proposed to make social networks resilient to the loss of individuals [30]. Using a metric that compares these features, the small-world coefficient [31], we found that seasonal small-world coefficients for the last 10 years were all significantly greater than 1 (electronic supplementary material, figure S2G; one-sample *t*‐test, *p* < 0.001, *t* = 26.13), indicating higher levels of clustering relative to path lengths compared to randomized control networks. This may explain the resilience of the barn’s social networks to both the predation events and RFID box removal that occurred in 2021. Taken together, these results characterize a wild mouse population with seasonally fluctuating small-world networks in which social groups are largest in winter and autumn, smallest in spring and summer, and females play a central role in group connectivity.

### (c) Vocal communication in wild house mice is seasonal

Acoustic communication has been proposed to play an important role in social group dynamics across mammals, although a majority of studies characterizing this role have focused on primates [4]. The seasonal fluctuations described above thus afforded an opportunity to ask what role acoustic communication plays in the social dynamics of wild house mice. To do this, we deployed AudioMoths [6] to record vocalizations in RFID boxes approximately once every two weeks between August 2022 and November 2023 (electronic supplementary material, figure S1A). For each deployment, we collected simultaneous, two-day long recordings from four boxes (one recording per box), resulting in 144 recorded boxes comprising 6594 total hours of audio (electronic supplementary material, figure S1B–D, electronic supplementary material, figure S3A,B). We then annotated vocalizations in a subset of these recordings, confirming that AudioMoths could detect the two primary vocalization types house mice make: squeaks and ultrasonic vocalizations (‘USVs’; electronic supplementary material, figure S3C,D).

**Figure 4.**
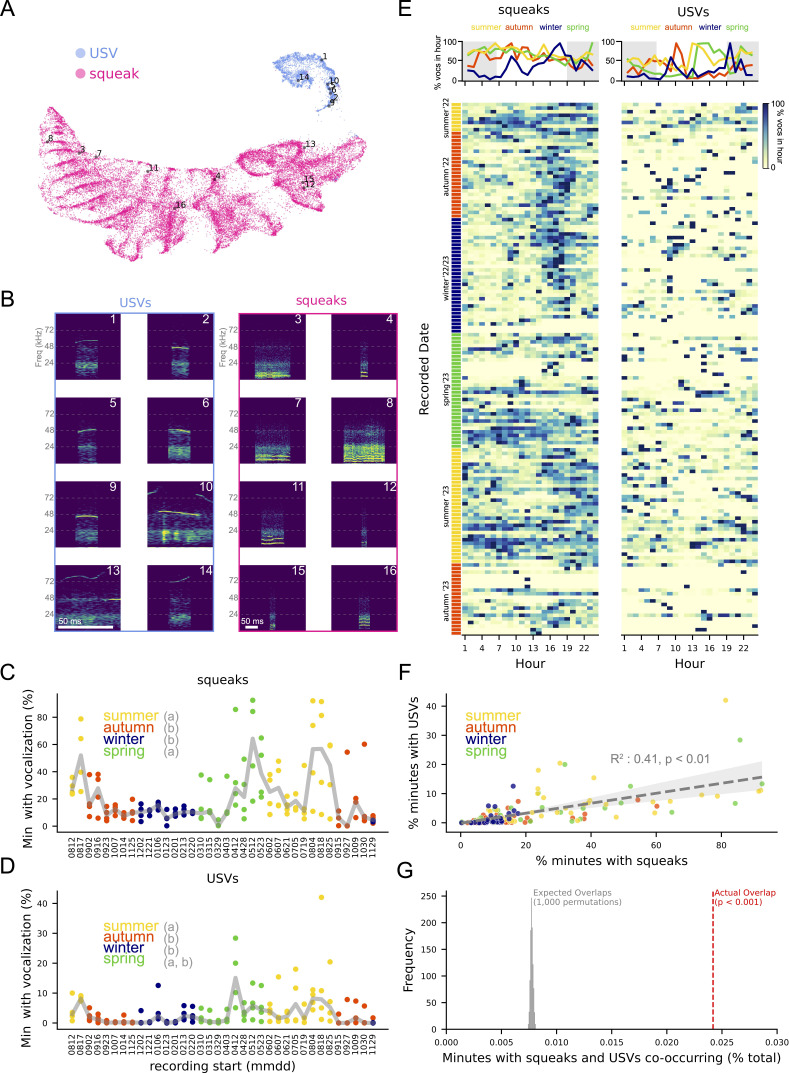
Season-specific patterns of vocalization. (A) UMAP embedding of 33 800 spectrograms sampled randomly from detected squeaks and USVs. Numbers match dots to spectrograms in (B). (B) Example spectrograms from A. Scale bars: 50 ms. (C) Percentage of recorded minutes with at least one squeak versus recording start date (mmdd). ANOVA: percentage of minutes with squeak ~ season, *p* < 0.001. Letters indicate seasons that differ significantly by Tukey post hoc test (*p* < 0.05). (D) Percentage of minutes with at least one USV versus recording start date. ANOVA: percentage of minutes with USV ~ season, *p* < 0.001. Letters indicate seasons that differ significantly by Tukey post hoc test (*p* < 0.05). (E) Number of squeaks (left) and USVs (right) by time of day. Top: average percentage of vocalizations per hour of day. Shading indicates night, using average yearly sunrise and sunset for Zürich. Bottom: heatmaps of data in top plot, each row is a single RFID box; colour corresponds to vocalizations per hour of day, normalized to the hour with the most vocalization. (F) Squeaks per recording versus USVs per recording, *R*^*2*^ = 0.41, *p* < 0.01, shading: 95% confidence interval. (G) Actual percentage of minutes containing both squeaks and USVs (red line) and percentage containing both in 1000 permutations of squeak timestamps relative to USV timestamps (grey), *p* < 0.01. See electronic supplementary material, S2 for complete ANOVA and Tukey tables.

Next, we sought to automatically identify squeaks and USVs in all 144 recordings. To do this, we used a temporal convolutional neural network (TCN) implemented by the software package Deep Audio Segmenter (DAS) [8], which has been shown to perform well on vocal segmentation tasks using relatively little training data and high signal-to-noise ratios [8] (see §4). We first trained and evaluated a TCN on the annotations described above (electronic supplementary material, figure S3E–K, vocal detection F1 score: 0.84; squeak label F1 score: 0.85, USV label F1 score: 0.78, see §4 for details), then used it to automatically detect and label vocalizations as either ‘squeak’ or ‘USV’. This revealed 1 366 171 total vocalizations, corresponding to approximately three vocalizations per minute from an average RFID box, with vocalizations labelled as squeaks being approximately 10 times more abundant than those labelled as USVs (1 260 452 squeaks versus 105 719 USVs). To gain confidence in these labels, we clustered their corresponding spectrograms in acoustic space with Uniform Manifold Approximation and Projection (UMAP, figure 4A). Spectrograms fell into one of two clusters that with few exceptions (e.g. USV no. 13) corresponded to predicted class labels (figure 4B).

To test the relationship between vocalization and features of seasonal social groups, we then asked if the amount of time mice spent vocalizing differed by recording season. We found that the percentage of recorded minutes containing at least one vocalization of either type was significantly affected by the season in which the recording was made (figure 4C,D; ANOVA: squeak-containing minutes ~ season, *p* < 0.001, F = 11.6; USV-containing minutes ~ season: *p* < 0.001, *F* = 8.4), with the majority of vocalization occurring in the summer and spring (91.6%). As we observe daily rhythms in box use by individual mice (figure 2B), we also asked if vocalization changed with time of the day. This was true for squeaks, but only in winter when they were most abundant in the afternoon and evening (figure 4E, left; ANOVA with Tukey post hoc test: squeaks ~ hour interval: *p* < 0.001, *F* = 15.6; hour intervals are 00.00−06.00, 'early morning'; 06.00−12.00, 'morning'; 12.00−16.00, 'afternoon'; and 16.00–24.00, 'evening'). For USVs, spring was the only season where we found an effect of time of day on vocalization, with more USVs produced in the afternoon than in the morning (figure 4E, right; ANOVA with Tukey post hoc test, USVs ~ hour interval: corrected *p* < 0.01, *F* = 4.9).

Squeaks and USVs have been proposed to signal distinct affective states in lab mice [32], suggesting they may be used in different social contexts. To test this in wild mice, we asked how often both vocalization types were detected in the same minute and compared this to the null hypothesis that production of each call type was independent of the other. We found that the amount of time mice spent producing USVs was positively correlated with that for squeaks (figure 4F; *R*-squared = 0.414, *p* < 0.001), and that the number of minutes containing both vocalization types was significantly higher than expected by chance (figure 4G; *p* < 0.01, *t* = 140.8). Thus, although squeaks and USVs may signal different affective states in some contexts, wild mice appear to produce them in close temporal association.

### (d) Vocalization is associated with the presence of pups

These findings indicate a seasonal correlation between vocal communication and wild mouse social groups: mice vocalized least during seasons characterized by large groups with less turnover (autumn and winter) and most during seasons characterized by small groups with more turnover (spring and summer). At shorter time scales, however, vocalization was highly variable across RFID boxes, even those recorded simultaneously (e.g. figure 4C,D). To identify sources of this variation, we next tested the relationship between vocalization and features of mouse social groups calculated on the time scale of two-day recordings.

To do this, we considered four hypotheses about the relationship between group composition and vocalization, and compared them using the Akaike information criterion (AIC) [33]: first, that vocalization is seasonal but not otherwise related to box occupants; second, that vocalization is seasonal and depends on the number of adult mice using the box; third, that it is seasonal and depends on the proportion of males to females using the box; and fourth, that it is seasonal and depends on the number of pups discovered in the box at the end of the recording.

**Figure 5.**
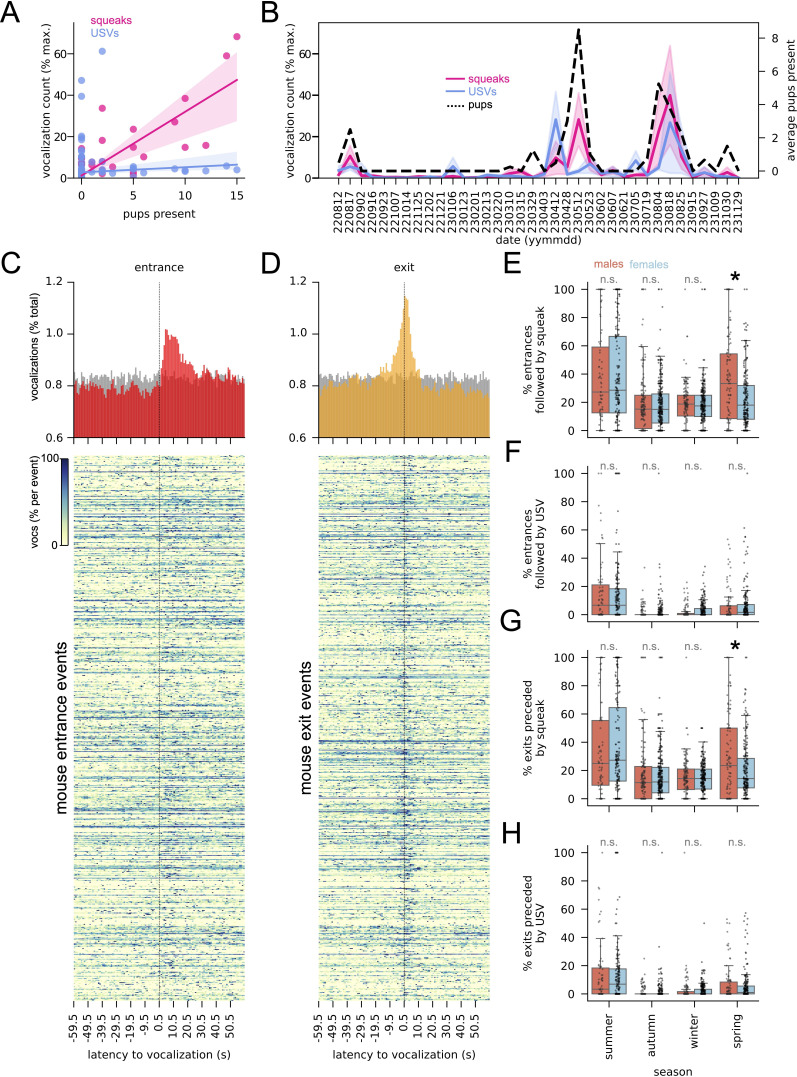
Vocalization is associated with pups and changes in box occupancy. (A) Number of pups found at the end of each recording versus squeaks or USVs detected in that recording. Shading: 95% confidence intervals. (B) Average squeaks and USVs recorded (as a percentage of maximum detected for each vocalization type) and average number of pups found in recorded boxes by recording date. Lines represent average per recording date. Shading for vocalizations: 95% confidence intervals. (C) Top: vocalizations aligned to box entrances (percentage total vocalization in each time bin). Bottom: each entrance in top plot with at least one vocalization preceded or followed by any vocalization within 60 seconds. Colour corresponds to percentage of vocalizations per time bin relative to bin with the most vocalizations in each row. (D) Top: vocalizations aligned to box exits (percentage total vocalization in each time bin). Bottom: data in top plot by exit, as in C. Colour as in C. (E) Percentage of entrances followed within 60 seconds by at least one squeak. ANOVA followed by Tukey post hoc test. Percentage entrances ~ season × sex: season *p* < 0.001, F = 47.8; sex *p* < 0.05, F = 5.7; season × sex *p* < 0.01, F = 5.6. Seasons with significant sex differences are starred. (F) Percentage of entrances followed within 60 seconds by at least one USV. ANOVA followed by Tukey post hoc test. Percentage entrances ~ season × sex: season *p* < 0.001, F = 67.6; sex n.s., season × sex n.s. (G) Percentage of exits preceded within 60 seconds by at least one squeak. ANOVA followed by Tukey post hoc test. Percentage entrances ~ season × sex: season *p* < 0.001, F = 53.5; sex n.s.; season × sex *p* < 0.05, F = 3.3. Seasons with significant sex differences are starred. (H) Percentage of exits preceded within 60 seconds by at least one USV. ANOVA followed by Tukey post hoc test. Percentage entrances ~ season × sex: season *p* < 0.001, F = 61.0; sex n.s.; season × sex n.s. See electronic supplementary material, S2 for complete ANOVA and Tukey tables.

The model in which vocalization depends on season and number of pups had the lowest AIC score for both squeaks and USVs. For squeaks, it also explained the most variance in vocalization counts, while for USVs the model in which vocalization depends on season and sex ratio did (figure 5A; electronic supplementary material, S3, table S1). Consistent with AIC scores, the average number of squeaks and USVs per deployment closely tracked the number of pups discovered in each recorded RFID box during each of the two waves of pups that occurred in 2023, one in early spring and one in late summer (figure 5B). While there are other, smaller peaks in vocalization that may correspond to non-pup related social interactions (possibly mating), these results suggest that pups are a strong predictor of vocal behaviour in wild house mice.

### (e) Vocalization is associated with box entrances and exits

Together, these analyses indicate that vocalization is correlated with features of mouse social groups on season- and days-long timescales. However, social interactions between individual mice typically occur on the time scale of seconds or shorter. While we did not collect video data to directly link such interactions with vocal events, RFID generated timestamps did allow us to ask if vocalization was associated with box events that change social group composition, i.e. entrances and exits. We found that 42% of all entrances and exits contained at least one vocalization within a two-minute window centred on the event (USV: 12.2%; squeak: 39.9%), and that vocalizations were closely associated in time with these events (figure 5C, red, and 5D, orange), trends we did not observe in simulated datasets in which vocalization times were randomized (figure 5C,D, grey; see electronic supplementary material, S1 for randomization details). We also found that the percentage of entrances and exits preceded or followed by at least one vocalization depended on season (squeaks: figure 5E,G; USVs: figure 5F,H; ANOVA with Tukey post hoc tests, *p* < 0.01; see figure 5 legend and electronic supplementary material, figure S2 for details). For squeaks this also depended on sex, with male entrances (figure 5E) more often followed by squeaks than those of females, and male exits (figure 5G) more often preceded by squeaks, an effect we only observe in spring (ANOVA with Tukey post hoc tests, *p* < 0.01; see figure 5 legend and electronic supplementary material, figure S2 for details).

### (f) Vocalization is correlated with how much time mice spend together

**Figure 6.**
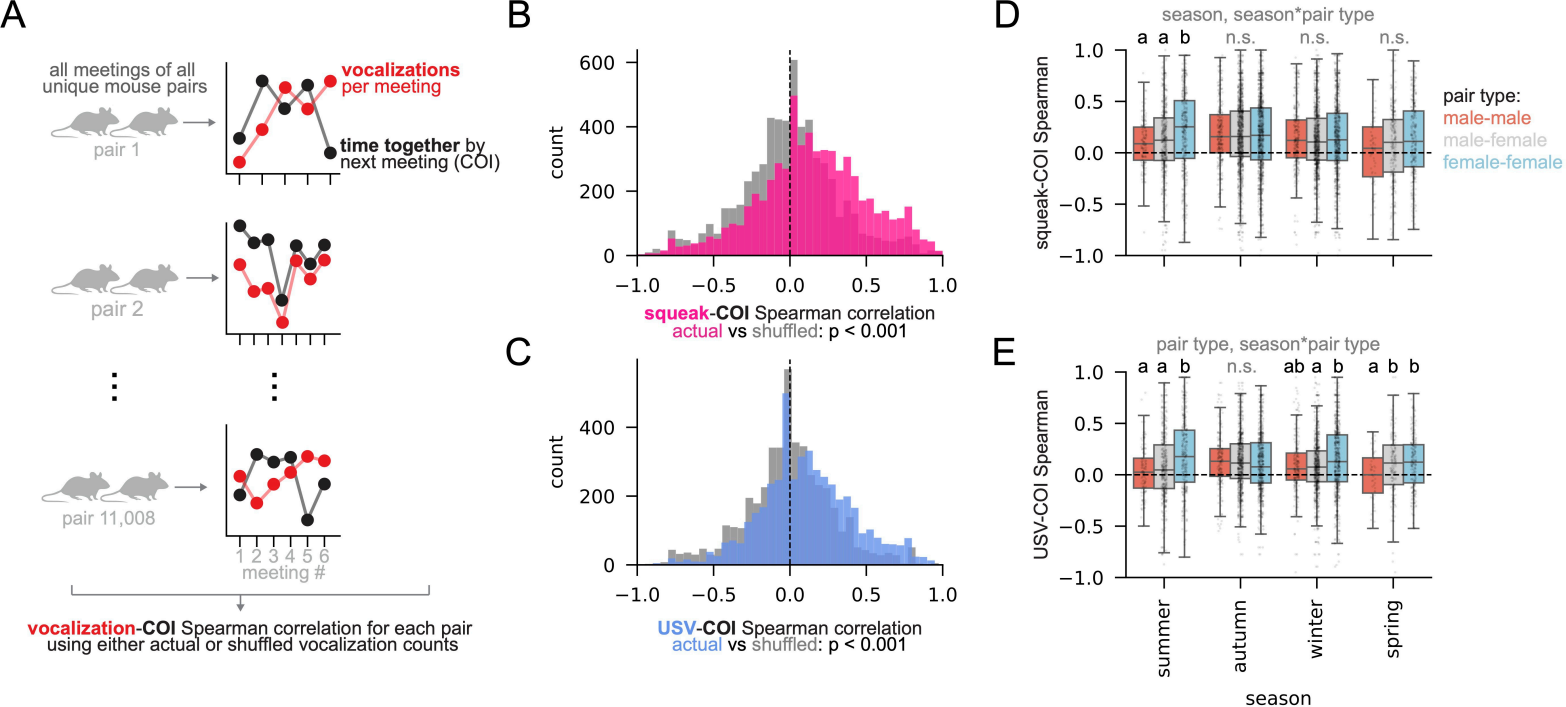
Vocalization is correlated with how much time mice spend together. (A) Analysis in panels B and C: for each unique mouse pair, Spearman correlation coefficients were calculated between vocalization counts recorded during each meeting of that pair and normalized time spent together (COI) by the start of the next meeting. Distributions of these correlations coefficients were then compared to coefficients generated from the same analysis, but with vocalization counts shuffled relative to COI values for each pair (B) Spearman correlation coefficients for actual squeaks versus time spent together (magenta) compared to shuffled controls (grey). Student’s *t*‐test, *p* < 0.001. See electronic supplementary materials, S1 for shuffling details. (C) Spearman correlation coefficients between actual USVs and time spent together (blue) compared to shuffled controls (grey). Student’s *t*‐test, *p* < 0.001. (D) The same correlation coefficients in panel B, split by sex and season. ANOVA followed by Tukey post hoc test for each season: Spearman’s ρ ~ season × pair_type. Significant ANOVA predictors are listed above plot. Letters indicate significantly different pair types within seasons. (E) The same correlation coefficients in panel C split by sex and season. ANOVA followed by Tukey post hoc test for each season: Spearman’s ρ ~ season × pair_type. Significant ANOVA predictors are listed above plot. Letters indicate significantly different pair types within seasons. See electronic supplementary material, S2 for complete ANOVA and Tukey Tables.

Vocalization in wild house mice is thus correlated with features of social groups at long (seasonal), medium (days), and short (seconds) time scales. If vocalization truly mediates social dynamics, however, vocal behaviours during a given social interaction should also be correlated with features of future interactions. To test this, we calculated Spearman correlation coefficients (ρ) between two time series for each unique pair of mice that spent time together in an audio-recorded box: first, the number of vocalizations they experienced during a given meeting in the focal (recorded) box; second, the normalized amount of time they had spent together in any box by the start of their *next* meeting in the focal box (i.e. their ‘co-occupancy index’ or COI; figure 6A; see electronic supplementary material, S1 for detail). We found that the amount of vocalization during a meeting in a given box was positively correlated with the amount of time mouse pairs had spent together by the start of their next meeting in that box (figure 6B, magenta (squeak), and 6C, blue (USV): actual correlations; grey: correlations from shuffled data; Student’s *t*‐test squeak: *p* < 0.001, t = 130.8; USV: *p* < 0.001, t = 76.7; see electronic supplementary material, S1 for detail). Thus, vocalization during social interactions is on average positively correlated with the duration of future interactions. These correlations were also highly variable, depending on season, sex, and the interaction between the two (figure 6D, squeaks: ρ ~ season × pair_type, where pair type is male–male, male–female or female–female; season *p* < 0.001, F = 6.8; pair_type n.s.; season × pair_type *p* < 0.01, *F* = 3.1; figure 6E, USVs: ρ ~ season × pair_type; season n.s.; pair_type *p* < 0.001, *F* = 9.3; season × pair_type *p* < 0.001, *F* = 5.2.). Taken together, these results suggest that vocal behaviours contribute to season- and sex-specific social dynamics in wild mice.

## 3. Discussion

Using automated tracking, we characterize social interactions in a population of wild house mice over the course of ten years. Consistent with previous work examining shorter time scales [24], we find that mice live in seasonal social groups in which females play a central role. In addition, we find that vocalization is correlated with features of these groups on season-, days- and seconds-long time scales. These results provide insight into the social structure of wild mouse populations and the role that vocal behaviours may play in contributing to that structure.

Our approach focuses on free-living, wild mice exposed to natural variation in environmental variables. As a result, the variance in our datasets is high. A complementary strategy is to focus on small populations over short timescales during which environmental variables are relatively stable. For example, recent studies have used RFID tracking in tens of rewilded laboratory mice exploring outdoor enclosures over periods of days during summer months. In these short-term experiments, male mice quickly established and defended territories at the exclusion of other males, and strain-specific female behaviours drove population-wide social structure [34,35].

Our findings in wild mice are generally consistent with these results. For example, males in our study population exhibited stronger box preferences than females, suggesting the establishment of male-specific territories, and overall females were more central in social networks than males, suggesting a larger role in shaping those networks. However, monitoring large populations in natural environments further allowed us to observe the effect of season on these behaviours. While males exhibited stronger box preferences than females across all seasons, all mice had stronger preferences in summers than winters. In addition, females spent more time using RFID boxes overall than males, a difference that was most dramatic in spring. We hypothesize that seasonal changes in both temperature and sex-specific reproductive behaviours shape these patterns. Future work will use temperature and weather data collected at the barn to disentangle contributions of environmental and biological variables to seasonal social dynamics.

Consistent with laboratory studies, we find that the vocalizations produced by free-living, wild house mice fall into one of two categories: USVs and low-frequency squeaks. While most laboratory studies have focused on USVs, we find that squeaks outnumber USVs in our dataset. This difference is probably due to an underestimation of USV counts, as AudioMoths are less sensitive at high frequencies [6], and to the fact that USVs are quieter than squeaks and thus less likely to be detected by our model (e.g. electronic supplementary material, figure S3K). While we are probably overestimating the difference between squeak and USV counts for these reasons, squeaks nonetheless comprise a large proportion of the vocalizations produced by mice in wild populations. In addition, we find that they occur in close temporal association with USVs more often than expected by chance. Although we do not know the identity of vocalizing mice, we hypothesize these associations result from contexts where the simultaneous use of both vocalization types conveys social information. For example, squeaks and USVs have been reported to occur in close temporal association in late stages of copulation [36], and low-frequency ‘wriggling’ calls produced by non-nursing pups may occur close to isolation induced pup USVs [18].

Tracking vocal behaviours over long time scales revealed that they are seasonal, with the majority of vocalization occurring in the spring and summer when social groups are smallest and most dynamic. Thus, social vocalization in wild mice is not strictly correlated with adult group size. Rather, we find that the presence of pups is a strong predictor of vocal communication. While we are unable to identify individual vocalizers in our recordings, a recent study of Mongolian gerbils found that removal of pups from social groups resulted in decreased vocalization [37], suggesting that pups, either by producing vocalizations (e.g. during isolation, handling or hunger states [32]) or triggering them in others (possibly during nest defense from infanticidal individuals [38]), are an important source of vocal communication in rodents.

Two key limitations of our study are that we can’t align vocal events to specific social behaviours using video data, and we cannot experimentally test the effect of specific vocalizations on social dynamics. Nonetheless, we do observe a close temporal association between vocalization and events (box entrances and exits) that change group membership. In addition, we find that, on average the amount of time pairs of mice spend together is positively correlated with how many vocalizations they experience together, and that these correlations depend on season and sex. We hypothesize that these sex differences result from sexually dimorphic roles for acoustic communication in house mice. For example, males may be more likely to use acoustic communication in the context of competition (e.g. for access to females in an RFID box) while females are more likely to vocalize within groups of other females with whom they are rearing pups [39]. Future studies will use acoustic playback experiments and video recording to directly test these hypotheses.

Short-term laboratory studies are essential to understand the genetic and neurobiological underpinnings of social behaviours. However, the amount of experimental control in these studies also limits their ability to describe social behaviours in the natural contexts in which they evolved to function. Technologies for long-term, passive monitoring of free-living populations now make it possible to fill this gap by capturing aspects of sociality that cannot be observed in the laboratory. Using these tools, this work lays a foundation for large-scale mapping between acoustic features of vocal signals and the social contexts in which those signals have meaning in natural populations.

## 4. Methods

### (a) Data collection

#### (i) Study population

We studied wild house mice living in a 72 m^2^ barn approximately 20 km from Zürich, Switzerland [40]. The barn contains *ad libitum* food (1 : 1 mix of ‘Hafer flockiert’, UFA AG, 3360 Herzogenbuchsee, Switzerland and ‘Meerschweinchen und Hamster Futter’, Landi Schweiz AG 3293 Dotzigen, Switzerland), water and cylindrical boxes (‘RFID boxes’, 15 cm tall, 15 cm diameter), each with a single entrance tunnel. Audio recordings were approved under permit ZH076/2022 by the Veterinary Office of Canton Zürich. RFID tracking between 2013 and 2023 was approved under permits ZH051/2010, ZH056/2013, ZH091/2016 and ZH098/2019.

#### (ii) Whole population censuses

Each mouse in the barn was caught bi-monthly, weighed and checked for an RFID tag before being returned to the approximate location where it was caught. Mice weighing 17.5 g or more with no tag were injected subcutaneously with one. See electronic supplementary material, S1 for details.

#### (iii) RFID tracking

Box use was monitored using an RFID system described previously [40]. The circular entrance tunnel of each box was fitted with two RFID readers connected to a computer inside the barn. RFID detections were logged automatically by this computer, then transmitted every 48 h to a remote database. See electronic supplementary material, S1 for details.

#### (iv) Audio recording

Acoustic analyses were performed on recordings from four AudioMoths (hardware version 1.2.0, firmware version 1.8.0). During each deployment, AudioMoths were left on top of haphazardly chosen RFID boxes and allowed to record at a sampling rate of 192 000 Hz with a 5 kHz high pass filter and ‘Medium’ gain. All other parameters were default, including the duty cycle of 55 s recording/5 s pause. One ‘minute’ in the context of our acoustic analyses thus corresponds to 55 s of audio. See electronic supplementary material, S1, for more detail.

#### (v) Aligning AudioMoth and RFID system clocks

We used an acoustic chime to account for differences between the onboard clock of each AudioMoth and the clock of the barn’s RFID system. At the start and end of each box recording, we passed a special ‘test’ transponder through the box’s outer RFID antenna. This transponder was designed to simultaneously generate a timestamp logged by the barn RFID system and a sound event (a chime from the barn computer), which was recorded by the AudioMoth. We manually assigned a timestamp to each recorded chime using the AudioMoth clock, identified the corresponding test transponder timestamp in the RFID database, and then adjusted vocalization timestamps according to the deviation between these two, assuming linear drift between AudioMoth and RFID clocks.

### (b) Data analysis

#### (i) Social network construction

We constructed social networks with python-igraph (v 0.11.2) [41] using default parameters. Communities were identified with python-louvain (v 0.16) [42] using default parameters. See electronic supplementary material, S1 for details.

#### (ii) Calculation of social network metrics

We calculated network modularity using python-louvain (v 0.16) [42]. We calculated node density, degree centrality, and clustering coefficients using networkX (v 3.2.1) [43]. See electronic supplementary material, S1, for details.

#### (iii) Annotation of acoustic data for neural network training

We used DAS (v 0.28.1) [8] to manually annotate the start and stop time of 2137 squeaks (197 s) and 2172 USVs (112 s) recorded during the summer, autumn, and winter of 2022. All annotations are included in the Dryad repository associated with this manuscript. See electronic supplementary material, S1, for more detail.

#### (iv) Neural network architecture and training

We trained a temporal convolutional neural network (deep audio segmenter’s tcn_stft model [8], no pre-training) on raw audio chunks consisting of 4096 samples each, equivalent to 21.3 ms, about half the length of the average USV in our annotations. See electronic supplementary material, S1 and [8] for more details.

#### (v) Neural network evaluation

Models were evaluated on their ability to label 21.3 ms chunks of audio as ‘cry’, ‘USV’ or ‘noise’ (i.e. non-vocal) using a 60%/20%/20% train/test/validation split within each annotated wav file (at the time of training, ‘cry’ was used as the label for vocalizations that we refer to here as ‘squeaks’). Similarly, vocal detection was evaluated as the ability to label 21.3 ms chunks of audio as vocal (‘squeak’ or ‘USV’) or ‘noise’ (non-vocal). Evaluation metrics were F1, precision and recall, as defined below:


$$\begin{array}{rl} & precision\;=\#\;correct\;labels\;/\;total\;predicted\;chunks\;with\;this\;label\\ & recall\;=\#\;correct\;labels\;/\;total\;actual\;chunks\;with\;this\;label\\ & F1=2\;/\;(1/precision\;+\;1/recall) \end{array}$$

#### (vi) Vocal segmentation

We detected and labelled vocalizations as squeak or USV with the predict method of the DAS package [8]. This returns an array of three probabilities, one for each possible label (‘squeak’, ‘USV’, ‘noise’), to chunks of audio 4096 samples in length (21.3 ms). See electronic supplementary material, S1, for more detail.

#### (vii) Spectrogramming and UMAP

UMAP embeddings were generated with the umap.fit_transform method of umap-learn python package [44] using default settings. See electronic supplementary material, S1 for detail.

#### (viii) Statistical modelling

Vocalization counts were analysed with the Python package statsmodels (v 0.14.4) [45] using generalized linear models (GLM) with a log link function and negative binomial distribution. A negative binomial distribution was chosen because counts were over-dispersed. For each model, an alpha parameter was chosen that minimized the model’s negative log-likelihood. We chose not to include vocalization timestamps, individual ID or box ID as random effects, as the number of mice using a given box is highly variable over time, and boxes are highly variable in their unique occupants. For non-count data, analysis of variance (ANOVA) was used to test for the effect of either season alone or a combination of season and sex on response variables, assuming a type I error rate of 5%. Tukey’s post hoc tests were used when ANOVAs indicated a significant effect (*p* < 0.05). ANOVA and Tukey post hoc tests were implemented using the anova_lm and tukeyhsd methods of statsmodels (v 0.14.4). See electronic supplementary material, S2 and S3 for tables corresponding to each statistical test performed in this study.

## Data Availability

All data required to reproduce analyses can be found on Dryad [46]. All code required to reproduce analyses can be found on GitHub [47]. Please see the README files associated with these repositories for detailed explanations of their contents and how to use them. All code required to reproduce analyses is also archived in Zenodo [48]. Supplementary material is available online [49].

## References

[1] Pasch B, Bolker BM, Phelps SM. 2013 Interspecific dominance via vocal interactions mediates altitudinal zonation in Neotropical singing mice. Am. Nat. **182**, E161–E173. (10.1086/673263) 24107377

[2] Liao CC, Magrath R, Manser M, Farine D. 2024 The relative contribution of acoustic signals versus movement cues in group coordination and collective decision-making. Phil. Trans. R. Soc. B **379**, 20230184. 38768199 10.1098/rstb.2023.0184 PMC11391321

[3] Martin M, Gridley T, Elwen S, Charrier I. 2022 Early onset of postnatal individual vocal recognition in a highly colonial mammal species. Proc. R. Soc. B **289**, 20221769. (10.1098/rspb.2022.1769) PMC9727656 36475443

[4] Xie B, Brask J, Dabelsteen T, Briefer E. 2024 Exploring the role of vocalizations in regulating group dynamics. Phil. Trans. R. Soc. B **379**, 20230183. 38768197 10.1098/rstb.2023.0183 PMC11391291

[5] Fisher JT. 2023 Camera trapping in ecology: a new section for wildlife research. Ecol. Evol. **13**, e9925. (10.1002/ece3.9925) 36937062 PMC10018383

[6] Hill AP, Prince P, Snaddon JL, Doncaster CP, Rogers A. 2019 AudioMoth: a low-cost acoustic device for monitoring biodiversity and the environment. HardwareX **6**, e00073. (10.1016/j.ohx.2019.e00073)

[7] Mathis A, Mamidanna P, Cury KM, Abe T, Murthy VN, Mathis MW, Bethge M. 2018 DeepLabCut: markerless pose estimation of user-defined body parts with deep learning. Nat. Neurosci. **21**, 1281–1289. (10.1038/s41593-018-0209-y) 30127430

[8] Steinfath E, Palacios-Muñoz A, Rottschäfer JR, Yuezak D, Clemens J. 2021 Fast and accurate annotation of acoustic signals with deep neural networks. eLife **10**, 68837. (10.7554/elife.68837) PMC8560090 34723794

[9] Cohen Y, Nicholson DA, Sanchioni A, Mallaber EK, Skidanova V, Gardner TJ. 2022 Automated annotation of birdsong with a neural network that segments spectrograms. eLife **11**, e63853. (10.7554/elife.63853) 35050849 PMC8860439

[10] Vélez J, McShea W, Shamon H, Castiblanco‐Camacho PJ, Tabak MA, Chalmers C, Fergus P, Fieberg J. 2023 An evaluation of platforms for processing camera‐trap data using artificial intelligence. Methods Ecol. Evol. **14**, 459–477. (10.1111/2041-210x.14044)

[11] Zipple MN, Vogt CC, Sheehan MJ. 2023 Re-wilding model organisms: opportunities to test causal mechanisms in social determinants of health and aging. Neurosci. Biobehav. Rev. **152**, 105238. (10.1016/j.neubiorev.2023.105238) 37225063 PMC10527394

[12] Crowcroft P. 1966 Mice all over. London, UK: Foulis.

[13] Berry RJ. 1970 Natural history of the house mouse. Field Studies **3**, 219–262.

[14] Vogt C. 2023 Spatial and social structure of rewilded laboratory mice. bioRxiv 2022.04.19.488643. (10.1101/2022.04.19.488643)

[15] König B, Lindholm AK. 2012 The complex social environment of female house mice (*Mus domesticus*). In Evolution of the house mouse (eds M Macholan, SJE Baird, P Munclinger), pp. 114–134. Cambridge, UK: Cambridge University Press. (10.1017/cbo9781139044547.007)

[16] Musolf K, Penn DJ. 2012 Ultrasonic vocalizations in house mice: a cryptic mode of acoustic communication, pp. 253–277. Cambridge, UK: Cambridge University Press. (10.5167/UZH-71386)

[17] Mai L, Inada H, Osumi N. 2023 Whole‐brain mapping of neuronal activity evoked by maternal separation in neonatal mice: an association with ultrasound vocalization. Neuropsychopharmacol. Rep. **43**, 239–248. (10.1002/npr2.12337) 37128179 PMC10275283

[18] Ehret G, Bernecker C. 1986 Low-frequency sound communication by mouse pups (Mus musculus): wriggling calls release maternal behaviour. Anim. Behav. **34**, 821–830.

[19] Ziobro P, Woo Y, He Z, Tschida K. 2024 Midbrain neurons important for the production of mouse ultrasonic vocalizations are not required for distress calls. Curr. Biol. **34**, 1107–1113.(10.1016/j.cub.2024.01.016) 38301649 PMC11696906

[20] Egnor SR, Seagraves KM. 2016 The contribution of ultrasonic vocalizations to mouse courtship. Curr. Opin. Neurobiol. **38**, 1–5. (10.1016/j.conb.2015.12.009) 26789140

[21] Lopes PC, König B. 2016 Choosing a healthy mate: sexually attractive traits as reliable indicators of current disease status in house mice. Anim. Behav. **111**, 119–126. (10.1016/j.anbehav.2015.10.011)

[22] Hofer MA, Shair HN, Brunelli SA. 2002 Ultrasonic vocalizations in rat and mouse pups. Curr. Protoc. Neurosci. Chapter 8, Unit 8.14. (10.1002/0471142301.ns0814s17) 18428567

[23] Tschida K, Michael V, Takatoh J, Han BX, Zhao S, Sakurai K, Mooney R, Wang F. 2019 A specialized neural circuit gates social vocalizations in the mouse. Neuron **103**, 459–472.(10.1016/j.neuron.2019.05.025) 31204083 PMC6687542

[24] Liechti JI, Qian B, König B, Bonhoeffer S. 2020 Contact patterns reveal a stable dynamic community structure with fission-fusion dynamics in wild house mice. bioRxiv 2020.02.24.963512. (10.1101/2020.02.24.963512)

[25] Latham N, Mason G. 2004 From house mouse to mouse house: the behavioural biology of free-living Mus musculus and its implications in the laboratory. Appl. Anim. Behav. Sci. **86**, 261–289. (10.1016/j.applanim.2004.02.006)

[26] Evans JC, Lindholm AK, König B. 2022 Family dynamics reveal that female house mice preferentially breed in their maternal community. Behav. Ecol. **33**, 222–232. (10.1093/beheco/arab128)

[27] Gerber N, Auclair Y, König B, Lindholm AK. 2021 Population density and temperature influence the return on maternal investment in wild house mice. Front. Ecol. Evol. **8**, 602359. (10.3389/fevo.2020.602359)

[28] Testard C *et al*. 2021 Rhesus macaques build new social connections after a natural disaster. Curr. Biol. **31**, 2299–2309. (10.1016/j.cub.2021.03.029) 33836140 PMC8187277

[29] Evans JC, Liechti JI, Boatman B, König B. 2020 A natural catastrophic turnover event: individual sociality matters despite community resilience in wild house mice. Proc. Biol. Sci. **287**, 20192880. (10.1098/rspb.2019.2880) 32370672 PMC7282912

[30] Watts DJ, Strogatz SH. 1998 Collective dynamics of “small-world” networks. Nature **393**, 440–442. (10.1038/30918) 9623998

[31] Telesford QK, Joyce KE, Hayasaka S, Burdette JH, Laurienti PJ. 2011 The ubiquity of small-world networks. Brain Connect. **1**, 367–375. (10.1089/brain.2011.0038) 22432451 PMC3604768

[32] Ehret G. 2013 Sound communication in house mice: emotions in their voices and ears? In Evolution of emotional communication (eds E Altenmüller, S Schmidt, E Zimmermann), pp. 63–74. Oxford, UK: Oxford University Press. (10.1093/acprof:oso/9780199583560.003.0004)

[33] Symonds MRE, Moussalli A. 2011 A brief guide to model selection, multimodel inference and model averaging in behavioural ecology using Akaike’s information criterion. Behav. Ecol. Sociobiol. **65**, 13–21. (10.1007/s00265-010-1037-6)

[34] Zipple M, Vogt C, Sheehan M. 2024 Genetically identical mice express alternative reproductive tactics depending on social conditions in the field. Proc. R. Soc. B **291**, 20240099. (10.1098/rspb.2024.0099) PMC10950460 38503332

[35] Vogt CC *et al*. 2024 Female behavior drives the formation of distinct social structures in C57BL/6J versus wild-derived outbred mice in field enclosures. BMC Biol. **22**, 35. (10.1186/s12915-024-01809-0) 38355587 PMC10865716

[36] Finton CJ, Keesom SM, Hood KE, Hurley LM. 2017 What’s in a squeak? Female vocal signals predict the sexual behaviour of male house mice during courtship. Anim. Behav. **126**, 163–175. (10.1016/j.anbehav.2017.01.021)

[37] Peterson RE, Choudhri A, Mitelut C, Tanelus A, Capo-Battaglia A, Williams AH, Schneider DM, Sanes DH. 2024 Unsupervised discovery of family specific vocal usage in the Mongolian gerbil. bioRxiv 2023. (10.1101/2023.03.11.532197) PMC11649239 39680425

[38] Auclair Y, König B, Lindholm AK. 2014 Socially mediated polyandry: a new benefit of communal nesting in mammals. Behav. Ecol. **25**, 1467–1473. (10.1093/beheco/aru143) 25419087 PMC4235584

[39] Ferrari M, Lindholm AK, König B. 2019 Fitness consequences of female alternative reproductive tactics in house mice (Mus musculus domesticus). Am. Nat **193**, 106–124. (10.1086/700567) 30624110

[40] König B, Lindholm AK, Lopes PC, Dobay A, Steinert S, Buschmann FJU. 2015 A system for automatic recording of social behavior in a free-living wild house mouse population. Anim. Biotelemetry **3**. (10.1186/s40317-015-0069-0)

[41] Csardi G, Nepusz T. 2006 The Igraph software package for complex network research. InterJournal Complex Syst. **1695**, 1–9.

[42] Aynaud T. 2020 Python-louvain 0.16: Louvain community detection. See https://pypi.org/project/python-louvain/.

[43] Hagberg AA, Schult DA, Swart PJ. 2008 Exploring network structure, dynamics, and function using NetworkX. In Proceedings of the Python in Science Conference, pp. 11–15. SciPy.

[44] McInnes L, Healy J, Melville J. 2018 UMAP: uniform manifold approximation and projection for dimension reduction. arXiv arXiv:1802.03426. (10.48550/arXiv.1802.03426)

[45] Seabold S, Perktold J. 2010 Statsmodels: econometric and statistical modeling with Python. In Proceedings of the 9th Python in Science Conference, pp. 92–96. SciPy. (10.25080/majora-92bf1922-011)

[46] Jourjine N, Goedecker C, Konig B, Lindholm A. 2025 Vocal communication is seasonal in social groups of wild, free-living house mice [Dataset]. Dryad Digital Repository. (10.5061/dryad.kprr4xhfk)40527459

[47] Jourjine N. 2025 Vocal communication is seasonal in social groups of wild, free-living house mice. GitHub. See https://github.com/nickjourjine/wild-mus-vocal-ecology.10.1098/rspb.2025.099540527459

[48] Jourjine N. 2025 nickjourjine/wild-mus-vocal-ecology: v1.0.2 (v1.0.2). Zenodo. (10.5281/zenodo.15375546)

[49] Jourjine N, Goedecker C, König B, Lindholm AK. 2025 Supplementary material from: Vocal communication is seasonal in social groups of wild, free-living house mice. Figshare. (10.6084/m9.figshare.c.7840154)40527459

